# A Quantitative Analysis of Internal and External Loads in Aviation Firefighting Using a Simulated Scenario

**DOI:** 10.3390/healthcare13020097

**Published:** 2025-01-07

**Authors:** Bronia Glen, Jodie Wills, Rhiannon Campbell, Stuart Cormack, Paul Tofari, Brendan Parsey, Rohan Edmonds, Tim Doyle

**Affiliations:** 1 Biomechanics, Physical Performance, and Exercise Research Group, Faculty of Medicine, Health and Human Sciences, Macquarie University, Sydney, NSW 2000, Australia; bronia.hardingdavis@hdr.mq.edu.au (B.G.); jodie.wills@mq.edu.au (J.W.); rhiannon.campbell@mq.edu.au (R.C.); rohan.edmonds@mq.edu.au (R.E.); 2 Performance and Expertise Research Centre, Macquarie University, Sydney, NSW 2000, Australia; 3 Sports Performance, Recovery, Injury & New Technologies (SPRINT) Research Centre, Australian Catholic University, Melbourne, VIC 3000, Australia; stuart.cormack@acu.edu.au (S.C.); paul.tofari@acu.edu.au (P.T.); 4 School of Behavioural and Health Sciences, Australian Catholic University, Melbourne, VIC 3000, Australia; 5 Airservices Australia, Canberra, ACT 2600, Australia

**Keywords:** tactical, physical employment standards, safety, injury, job readiness

## Abstract

Background/Objectives: Aviation firefighting is a strenuous occupation that requires individuals to engage in intense physical activity amidst elevated stress levels and extreme environmental conditions. Despite this, there has been limited investigation regarding the internal and external loads associated with aviation firefighting tasks, which include hose dragging, stair climbing, casualty evacuation, and fire extinguishing in airports and aircrafts. The aim of this study was to examine the internal and external loads placed on aviation firefighters. By identifying these demands, this study seeks to inform the development of targeted training strategies, improve job safety, and lower the risk of musculoskeletal injuries. Methods: Sixteen Australian aviation firefighters (35.13 ± 8.2 years) were recruited and assigned specific roles to complete an aircraft firefighting scenario. Measures of heart rate (HR), oxygen consumption ($\dot{V}$O_2_), and rating of perceived exertion (RPE) were used to quantify internal load, while measures of completion time and distance travelled were used to quantify external load. Results: The median scenario completion time was 21 min (IQR = 5), with each role travelling a median distance of 245–541 m. During the scenario, median average HR values ranged between 61.1 and 72.0% HRmax and median maximal HR values ranged between 77.8 and 84.4% HRmax. As the only group to record $\dot{V}$O_2_, driver firefighters operated at a median average $\dot{V}$O_2_ of 49% of their $\dot{V}$O_2max_ and achieved a median maximal $\dot{V}$O_2_ of 78% of their $\dot{V}$O_2max_. Conclusions: This study effectively identified the task-specific internal and external loads associated with aviation firefighting, offering valuable insights for developing specific training protocols for firefighters to ensure appropriate physical capacity to perform their job roles safely.

## 1. Introduction

Firefighting is a physically demanding occupation involving bouts of high-intensity work performed under high stress and heat loads [1,2]. Firefighters are required to drag heavy hoses over distances, climb stairs/ladders, and perform casualty evacuations whilst wearing personal protective equipment (PPE) weighing between 15 and 25 kg [2,3,4]. Within the firefighting occupation, there are multiple specialisations, including structural, wildland, military, and aviation. Aviation rescue firefighters respond to fires and mitigate hazards within airports and surrounding areas. They are the first responders for fuel spills, engine/aircraft fires, emergency landings, wildland fires in airport surroundings, and evacuation of passengers and airport personnel [2,3]. However, there is limited research on the internal and external loads experienced specifically during aviation rescue firefighting (ARFF). External loads refer to objective measures which quantify the work performed (e.g., task completion time) and can be referred to as the stimuli which elicit intrinsic responses. Internal load encompasses the intrinsic response to external loads, including both physiological indicators (e.g., heart rate, HR) and subjective measures of effort (e.g., rating of perceived exertion, RPE) [5].

The mandatory use of PPE [2,3,4], including a 10–12 kg self-contained breathing apparatus (SCBA) required in smoke and fire environments [4,6,7], significantly increases both internal and external loads during firefighting, as demonstrated in multiple studies [8,9,10]. For example, PPE increased completion time in a wildland firefighting training task (walking 4.8 km with a 20 kg load) by 12% [8] and PPE/SCBA increased completion time in a metropolitan structural firefighting simulation (involving a stair climb, hose drag, forcible entry, and search and rescue) by 45% [9]. Additionally, RPE during physical tasks is significantly higher when wearing PPE compared to exercise clothes [4,9] and Marcel-Millet et al. [10] highlight how wearing an SCBA significantly increases HR and RPE compared to personal protective clothing alone. Quantifying loads, that is, the physical constraints and physiological demands, experienced by firefighters is important for developing safety regulations, physical fitness standards, and injury prevention measures, in order to minimise injury risk [11].

Several studies have investigated the cardiovascular strain of firefighting, with firefighting activities reportedly resulting in HR values between 60 and 90% of the maximum (%HRmax; maximum HR as a percentage of absolute HRmax) [12]. Rescue simulations have observed maximal HR values that reach 97% [13] and 96% of HRmax [14]. During flashover training, a scenario involving the rapid spread of fire caused by the sudden ignition of combustible material within an enclosed area [15], aviation firefighters have been observed reaching maximal heart rates of 95% HRmax [12,15]. Performing strenuous activity in extreme heat and smoke conditions places additional strain on cardiovascular and thermoregulation systems [12]. Firefighting in extreme temperatures (e.g., >300 °C) results in higher mean and maximum HR and longer HR recovery compared to firefighting in temperate conditions [12]. However, reporting internal load using heart rate (HR) has limitations due to the intermittent nature of firefighting and the challenges of relying solely on HR in hot environments. Studies have also shown that RPE scores are higher in hot (45 °C) and extremely hot (300 °C) firefighting conditions compared to temperate [12,16]. Hence, firefighting imposes a considerable cardiovascular burden, potentially exacerbated by elevated temperatures, which requires firefighters to maintain appropriate physical fitness to perform firefighting tasks safely.

The maximal volume of oxygen able to be utilised during physical activity ($\dot{V}$O_2max_) is routinely used to measure cardiovascular fitness and assess an individual’s physiological capability to perform a given physical task [4,17]. Previous studies have quantified the metabolic demand of firefighting tasks, recommending an optimal $\dot{V}$O_2max_ between 41 and 45 mL·kg·min^−1^ for firefighters to meet the external load demand [18,19]. Indeed, it has been suggested that firefighters with an aerobic capacity that does not match the demands of the job are between 1.4 and 2.2 more likely to incur an injury [20]. Fortunately, however, these same researchers found that an improvement in aerobic capacity can reduce injury occurrence by 14% [20]. Gledhill and Jamnik [19] suggest that for most tasks, firefighters work at 50–85% of their $\dot{V}$O_2max_, with 90% of tasks requiring a mean $\dot{V}$O_2_ of 23 mL·kg·min^−1^; the most demanding tasks require an average $\dot{V}$O_2_ of 41.5 mL·kg·min^−1^. However, whilst a mean $\dot{V}$O_2max_ of 49.5 ± 6.9 mL·kg·min^−1^ has been characterised for an aviation firefighter population [2], ARFF task-specific $\dot{V}$O_2_ requirements have not been assessed. Assessing $\dot{V}$O_2_ is an important tool to gauge the physiological load of distinct firefighting activities and indicate the appropriate fitness level essential for firefighters to meet the external load requirements and complete occupational tasks safely.

As contextual differences affect the activity profile of the task, firefighting research should address this specific specialisation of firefighting. However, most studies examining workload have focused on structural firefighters [7,21,22,23,24,25,26,27]. Recently, Jagim et al. [22] assessed the demands of an air management course to evaluate PPE tolerance, SCBA use, and firefighting performance in structural firefighters. The scenario involved a hose line advance, casualty evacuation, stair climb, ladder raise, and forcible entry, followed by laps of a course (stair climb, search, hoist, and walk) until SCBA air pressure decreased to 200 PSI. Measures of internal load included HR and training impulse (Bannister’s TRIMP), and estimated energy expenditure, with external load being quantified using GPS (to measure distance travelled). The task resulted in a mean HR of 87% HRmax and participants with a BMI < 25 kg/m^2^ had a greater work efficiency (measured in km·PSI^−1^·s), completed more laps of the course, and covered a greater distance compared to participants with a BMI > 25 kg/m^2^. More recently, Marciniak et al. [6] found that the internal workloads for structural firefighters are six times more demanding, both physiologically and perceptually, and are twice as cumulative compared to those encountered during medical and other fire emergency responses. Whilst the internal and external load of structural firefighting has been touched on in earlier research, the understanding of these loads specific to aviation firefighting is currently lacking.

Most recently, Skinner et al. [2] correlated physical fitness with performance in an ARFF emergency protocol, involving hose/dummy drags and stair climbs with an SCBA. Fast completion times were positively associated with aerobic capacity ($\dot{V}$O_2max_), anaerobic step test scores, and lean mass, and negatively associated with fat mass and body fat percentage [2]. However, whilst ARFF personnel were used in the study, the ARFF protocol did not involve aircraft/airport-specific structures [2]. Furthermore, the focus of the study was to correlate body composition and physical fitness attributes with task performance, rather than define the internal and external load associated with ARFF tasks [2]. Additionally, Windisch et al. [12] assessed performance in aviation firefighters by measuring HR, completion time, and air depletion during an occupational training exercise. The tasks involved a treadmill walk, ladder climb, rope hoist, tunnel crawl, and firefighting (five fires, including two unexpected flashovers) within a computer-controlled heated container. This allowed for an opportunity to respond to a staircase fire, but not an aircraft fire [12].

To date, limited research has explored the specific workload demands across firefighting roles, particularly within ARFF. Jagim et al. [24] highlight the variability in physical and physiological demands between operational and administrative roles, although their findings do not specifically address aviation-specific firefighting tasks. Similarly, Marciniak et al. [6] underscore the importance of understanding role-specific loads to optimise performance, reduce injury risk, and enhance training protocols across diverse firefighting contexts.

To our knowledge, no other studies have quantified the internal and external loads experienced by aviation firefighters when performing aviation-specific firefighting tasks. Furthermore, very few studies examine load differences based on specific firefighting roles, a crucial aspect considering the higher physical demands of active firefighting roles compared to organisational roles [23]. Thus, the aim of this study was to quantify the internal and external load during an aviation firefighting scenario. The scenario mimics real-world situations to inform physical preparation programmes that help equip firefighters for job demands, potentially reducing the injury risk associated with a mismatch between firefighter capabilities and task requirements.

## 2. Materials and Methods

### 2.1. Participants

Sixteen operational aviation firefighters (15 male, 1 female; age 35.13 ± 8.2 years; height 1.79 ± 1.0 m; body mass 87.6 ± 16.5 kg; BMI 27.1 ± 3.5 kg/m^2^; years of service 5.4 ± 4.2 years) were recruited from a single firefighting organisation to complete aviation-specific firefighting scenarios. The project was advertised only to this organisation, and participants volunteered and provided written informed consent. The research was approved by the Macquarie University Human Research Ethics Committee (REF: 52021993530114).

### 2.2. Procedures and Protocols

#### 2.2.1. Physical Testing

All physical testing was completed one day prior to scenario completion (Table 1) to obtain baseline strength and cardiovascular measurements. Height was measured using a portable stadiometer (Cescorf, Porto Alegre, Brazil) and body mass was measured on portable force plates (VALD ForceDecks; FDLite V.1, VALD Performance, Brisbane, QLD, Australia) using the VALD ForceDecks software (2.0.8587). The same force plates were used for jump and isometric testing as detailed below. A goniometer was used to measure knee, hip, and elbow angles.

Countermovement Jump (CMJ): Maximum jump height, in centimetres, was measured with participants performing countermovement jumps (CMJs). Participants were instructed to flex at the hip and knee to a self-selected depth and jump as high as possible, with hands on hips and feet shoulder-width apart [28], with each foot landing on a force plate. The best of three efforts was used for analysis.

Squat Jump (SJ): Three SJs were performed as per the previously described protocol [28] and maximum jump height (cm) was recorded. Participants were instructed to place their hands on their hips, hold a ~3 s squat at a self-selected depth, and jump as high as possible without a countermovement. The best of three efforts was used for analysis.

Isometric Mid-Thigh Pull (IMTP): Maximum lower-limb strength, measured as peak force (N), was obtained using an IMTP, performed according to procedures previously described [29]. Participants stood on VALD force plates, with their feet approximately hip-width apart, knees slightly in front of the bar, and thighs touching the bar. The bar height was adjusted such that knee and hip angles were between 125 and 145° and between 140 and 150°, respectively. Participants were instructed to apply maximum effort to pull the immobile bar vertically (locked in place with weights and secured with non-compliant straps). Wrist straps were used to fix participants’ hands to the bar to ensure grip strength was not a limitation when assessing maximal lower-body strength. After a standardised warm-up protocol, including sub-maximal efforts, participants completed one repetition, and the peak force was obtained.

Isometric Push-Up (IPU): Upper body strength, measured as peak force (N), was obtained using an IPU, where participants assumed a standard push-up position (elbow angle, 90°) and used maximum effort to push upwards on an immobile bar (pushing hands into VALD force plates) [30]. Following a standardised warm-up protocol, participants completed one maximal repetition and peak force was recorded.

Multi-Stage Fitness Test (MSFT): To predict maximal oxygen consumption and ascertain cardiovascular fitness, participants (*n* = 15) completed the MSFT [31]. Participants performed shuttle runs with auditory cues, progressively increasing in speed, over a 20 m distance, until volitional exhaustion. One participant (operator role) did not perform the beep test due to a lower-limb injury. HR was recorded during the beep test using a Polar H10 chest strap transmitter (Polar Electro Oy, Kempele, Finland), connected to a Garmin Instinct Solar watch (Garmin Ltd., Olathe, KS, USA).

#### 2.2.2. Scenario Testing

An aviation firefighting training scenario was developed involving an engine fire through extensive consultation with subject matter experts and directed by highly experienced fire commanders. Aircraft scenarios were completed by four groups of four ARFF firefighters, across two geographically different fire station locations. The scenario was conducted under two conditions, without fire (cold) and with fire (hot), to simulate variations in real-life training environments. Further to this, as the primary aim was not to compare the conditions, the hot condition in the current study was not intended to induce additional thermal stress, with only one lit fire in the hot condition. As such, data from both scenarios were grouped to provide a comprehensive analysis of the overall physical load experienced during firefighting activities. The testing schedule is shown in Table 1. Each participant was assigned an active firefighting job role in line with real-world positions (Table 2).

PPE: All participants wore standard firefighting PPE, including a heat-protective jacket, pants, boots, gloves, flash hood, helmet, and SCBA (13 kg).

GNSS data: Total distance travelled and average speed were measured via a global navigation satellite system (GNSS) unit (Apex, STATSports, Newry, Northern Ireland; 84 × 43 × 20 mm; 72 g), housed in a custom-designed tightly fitting harness, worn underneath the PPE detailed above.

HR monitoring: During the scenarios, participants wore a Polar H10 chest strap transmitter connected to a Garmin Instinct Solar watch, to continuously measure HR.

Oxygen consumption: The firefighter driver (DR) was determined to be the most physically demanding role, based on input from subject matter experts, and hence was fitted with a COSMED K5 wearable metabolic system (Cosmed, LLC., Rome, Italy).

Rate of perceived exertion: Approximately 10–30 min after the end of scenario testing, participants provided an RPE score, using the Borg CR100 scale [32], with 1.5 and 100 representing minimum and maximal effort, respectively.

**Figure 1:**
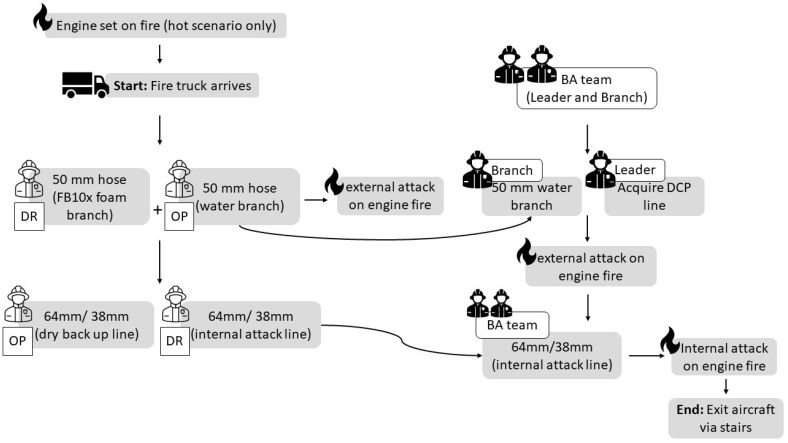
Aircraft scenario flow diagram. White shapes represent firefighting roles; grey boxes represent tasks. BA, breathing apparatus; DR, driver; OP, operator; DCP, dry chemical powder.

Aircraft scenario: The aircraft scenario required aviation firefighters to respond to a fire in a plane engine. Prior to the hot scenario, the engine and various locations within the craft were set on fire. For the external attack, the DR set up one 50 mm hose connected with an FB10x foam branch, and the OP pulled out a 50 mm water branch. The water branch was given to the BA team branch, and the BA team leader acquired a DCP hose line for a dual, external attack. During the external attack, the DR organised a 64 mm/38 mm internal attack line, which was passed to the BA team. The BA team advanced the internal attack line up the stairs, checked/sprayed the doorway, and entered the aircraft to internally attack the engine fire. The scenario was completed when the BA team exited the aircraft and fed the hose back down the stairs. The scenario commenced when the fire truck came to a stop and proceeded as outlined in Figure 1.

### 2.3. Data Analysis

#### 2.3.1. Baseline Measurements

Maximum jump height (cm), determined by the impulse–momentum relationship, was exported from the ForceDecks software for all CMJ and SJ trials. The maximum value for each participant was used for analysis. The eccentric utilisation ratio (EUR) was recorded as the ratio between CMJ and SJ height and indicates stretch shorting cycle (SSC) performance [33]. Peak, absolute force (N), and force relative to body weight achieved during the IMTP and IPU were also extracted from ForceDecks software (sampling frequency, 1000 Hz). Continuous HR was measured on the Garmin watch during the MSFT, with maximum HR recorded. HR has not been included for one participant due to non-completion of the MSFT. For one participant, HR was recorded with a watch rather than the Polar HR monitor. Predicted $\dot{V}$O_2max_ was calculated from the MSFT result using the formula from Ramsbottom and Brewer [34]:

$$\mathrm{Predicted}\;\dot{V}O_{2\max}=3.46\times \left(\frac{\mathrm{Beep}\;\mathrm{level}+\mathrm{Beep}\;\mathrm{shuttle}}{\mathrm{Beep}\;\mathrm{level}\times 0.4325+7.0048}\right)+12.2$$

#### 2.3.2. Scenario Measurements

Total distance, time taken to complete the scenario, and speed were recorded on the GNSS. Continuous HR was measured to calculate average and maximum HR. Maximal HR during the scenario was used to calculate %HRmax (maximum HR as a percentage of absolute HRmax, calculated from the MSFT). For some participants, maximum HR was incorrect or missing (due to equipment failure). Edwards’ TRIMP values were calculated using raw HR time-series data, converted to a percentage of each participant’s HRmax (%HRmax) [35]. The time spent in each HR zone (minutes) was multiplied by the zone number. HR zones, as previously defined [36], are presented in Table 3.

The TRIMP value is the sum of the points from each HR zone:

TRIMP (AU)=(time in zone 1×1)+(time in zone 2×2)+(time in zone 3×3)+(time in zone 4×4)+(time in zone 5×5)

For DR firefighters, the COSMED K5 device measured time spent using the device and the volume of oxygen consumed which was used to calculate average $\dot{V}$O_2_, $\dot{V}$O_2max_, and %$\dot{V}$O_2max_ (scenario $\dot{V}$O_2max_ as a percentage of $\dot{V}$O_2max_ estimated from MSFT).

### 2.4. Statistical Analysis

Descriptive statistics were calculated using Jamovi (Version 2.4.8) and graphs were generated using GraphPad prism software (v10.1.2; GraphPad Software, San Diego, CA, USA). Data violated assumptions of independence and normality. As such, nonparametric statistics were performed, accounting for the small participant sample size and individual variability in scenario performances and the results are presented as median and interquartile range (IQR). Significance testing was performed using GraphPad Prism software, with rank-based nonparametric Kruskal–Wallis tests and Dunn’s post hoc tests to assess differences among the four firefighting roles. Significance between roles was established at *p* < 0.05, and rank-based effect size statistics were calculated using an Epsilon-squared (ε2) test. The magnitude of the effect was categorised as follows: 0.01–<0.06 (small effect), 0.06–<0.14 (moderate effect), and >0.14 (large effect). In line with the primary aim of the study, and since the sole external fire in the hot condition was not intense enough to induce additional thermal stress beyond what the PPE provided, data from both conditions were grouped for statistical analysis. This grouping was deemed appropriate based on no statistically significant differences in distance travelled, RPE, TRIMP, and average/maximal HR between the hot and cold scenarios for each group (*p* < 0.05 for all hot/cold comparisons). This approach was taken to capture the cumulative effects of the training scenario across different environments rather than to compare the conditions themselves.

## 3. Results

Participant baseline cardiovascular and strength measurements are presented in Table 4.

### Aviation Scenario

**Figure 2:**
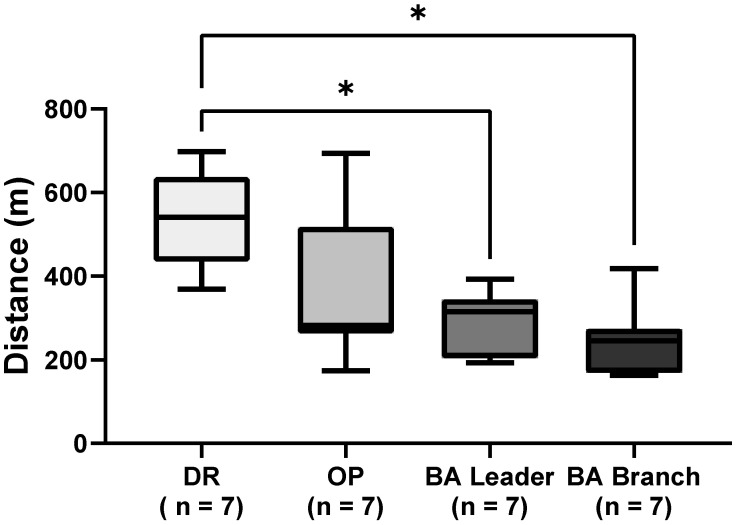
Total distance travelled during the aviation scenario. Median and 25th/75th percentiles are shown in each box plot, with whiskers representing the minimum and maximum values. Asterisks denote significance: * *p* < 0.05. DR, driver; OP, operator; BA, breathing apparatus.

The scenario took 17–27 min (median = 21 min, IQR = 5) to complete. The total distance travelled by each role during the aviation scenario is presented in Figure 2. A Kruskal–Wallis test was conducted to examine differences in distance travelled among the four groups. The test revealed a significant effect, H(3,N = 28) = 12.97, *p* ≈ 0.0047, with a large effect size, ε2 = 0.30. Dunn’s multiple comparisons post hoc tests showed that the DR group travelled significantly further (median = 541 m, IQR = 114.5) than the BA leader (median = 315 m, IQR = 107.5, adjusted *p* = 0.04) and BA branch groups (median = 245 m, IQR = 79, adjusted *p* = 0.004), while other comparisons were not statistically significant.

**Figure 3:**
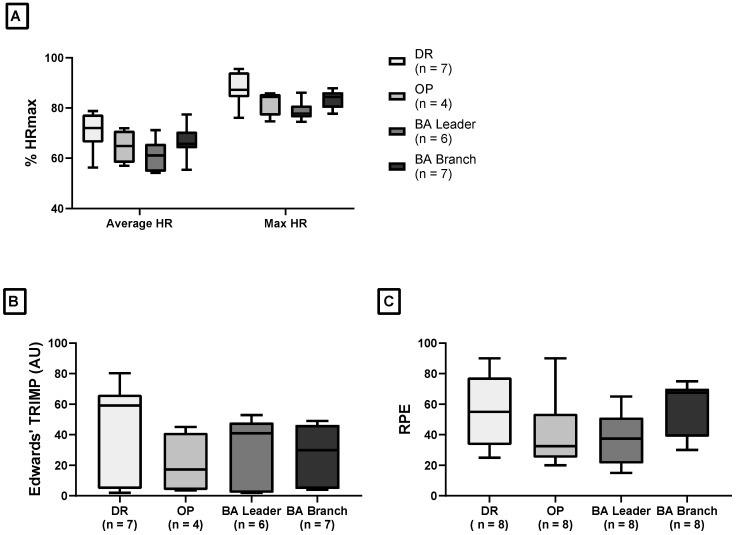
Internal load measures during the aviation scenario: (**A**) relative average and maximal HR (%HRmax), (**B**) Edwards’ TRIMP (AU) scores, and (**C**) RPE. Median and 25th/75th percentiles are shown in each box plot, with whiskers representing the minimum and maximum values. DR, driver; OP, operator; BA, breathing apparatus; TRIMP, training impulse; RPE, rate of perceived exertion.

Median average and maximal HR values as a percentage of HRmax (obtained from the MSFT) are presented in Figure 3A. DR firefighters (*n* = 7) had an average HR that ranged from 106 to 154 bpm. Similarly, average HR for OP firefighters (*n* = 6) ranged between 106 and 151 bpm. The BA leader group had an average HR between 99 and 148 bpm and the average HR for the BA branch group ranged from 99 to 153 bpm. Firefighters displayed maximal HR values of 77.8–87.2% HRmax (DR: median = 87.2%, IQR = 7.20; OP: median = 84.4%, IQR = 3.42; BA leader: median = 77.8%, IQR = 1.98; BA branch: 84.4%, IQR = 4.85). There were no significant differences (*p* > 0.05) in relative average (ε2 = 0.17) and maximal HR (ε2 = 0.23) values among the firefighting roles, as indicated by Kruskal–Wallis tests. Edwards’ TRIMP [34] and RPE scores for each role are presented in Figure 3B,C, respectively. Median TRIMP scores ranged from 17.2 AU (IQR = 28.9) for OP firefighters to 59.1 AU (IQR = 59.9) for DR firefighters. Median RPE scores for the four firefighting roles ranged from 32.5 (IQR = 26.3) for the OP group to 67.5 (IQR = 23.8) for the BA branch group. No significant differences (*p* > 0.05) were found in TRIMP (ε2 = 0) and RPE (ε2 = 0.04) scores across the firefighting roles, according to Kruskal–Wallis tests.

DR firefighters wore the wearable metabolic system whilst completing the aviation scenario, with average and maximal oxygen consumption data presented in Table 5.

## 4. Discussion

While there are considerable studies in the literature on structural and wildland firefighting, there is limited information about the unique role of aviation rescue firefighting. Since the internal and external load of aviation firefighters remains unexplored, the current study aimed to determine the internal and external load experienced during aviation-specific firefighting tasks. Developed by subject matter experts, the scenario aimed to replicate a real-world situation, requiring firefighters in various roles to collaborate and respond to a simulated hazard.

The aviation scenario studied in the current study was completed in 17–27 min. These times were comparable to the flashover training simulation (15.5 ± 1.2 min) completed by firefighters at Munich Airport [12] but longer than the 4.5 min ARFF emergency protocol employed by Skinner et al. [2]. Participants in the DR role covered the greatest distance and travelled significantly further than the BA leader and BA branch groups (541 m vs. 315 m vs. 245 m, respectively). This reflects role differences, where DR firefighters spend much of the scenario organising hoses, whereas the BA team only cover distance during the internal attack.

Relative maximal HR ranged between 77.8 and 87.2% HRmax, comparable to the 80% HRmax value attained by firefighters completing a rescue, search, and run task [13]. In the present study, relative HRmax was lower than observed by Windisch et al. [12], who showed that aviation firefighters working in high temperatures (300 °C) and smoke had an average HRmax above 95% for the last four minutes of the flashover training task. However, their task involved extinguishing five fires compared to just one fire in the present study.

DR firefighter TRIMP scores (median = 59.10) are comparable to those attained after 36 min of wildland fire suppression (mean = 54.9) [36] but lower than TRIMP values reported after a 25 min firefighting task loop (mean = 79.2) [22]. However, TRIMP values for firefighters in the OP (median = 17.20), BA leader (median = 40.95), and BA branch roles (median = 29.90) are lower than those presented in the current literature [22,36]. Low TRIMP values in the present study could be explained by a shorter scenario time or a less intense scenario (less time working at higher HR zones) compared to that employed by Jagim et al. [22]. Further, increased sample sizes would be useful in quantifying TRIMP scores reflective of aviation firefighting, as TRIMP scores varied greatly in the current study.

BA branch firefighters reported RPE scores corresponding to ‘very strong’ exertion (RPE = 67.5) and DR firefighters reported RPE scores corresponding to ‘strong’ exertion levels (RPE = 55) using the Borg CR100 scale [32]. RPE values for firefighters in both roles are comparable to those presented by Larsen et al. [16], who used the 6–20 point scale [37]. Ten wildland firefighters completing a debris raking task for 3 h had a greater RPE at 45 °C (15.6, ‘hard’) compared to 18 °C (12.6, ‘somewhat hard’) [16]. However, RPE scores in the current study showed considerable variability, as individuals perceive a given external load differently based on varying interpretations of task-relevant factors, reflecting the subjective nature of the scale. For example, participants in the DR role reported an RPE of 25–90. Whilst highly variable in the current study, quantifying internal load by means of an RPE scale offers valuable insight into an individual’s internal ability to cope with the external load of a task. The use of RPE to estimate physical effort [38] and quantify the internal loads of various aviation firefighting roles has merit, especially when the external loads of these tasks cannot be adjusted [38].

In our cohort, the median predicted $\dot{V}$O_2max_ (47.11 mL·kg·min^−1^) was greater than the suggested minimum for firefighters (41–45 mL·kg·min^−1^) [19]. Further, 87% of participants (*n* = 13) met the suggested minimum (>41 mL·kg·min^−1^), with 67% (*n* = 10) achieving a $\dot{V}$O_2max_ > 45 mL·kg·min^−1^ compared to 74% (*n* = 31) of ARFF participants in previous research [2]. However, despite 13% of participants not meeting the $\dot{V}$O_2_ requirements, all completed the ARFF protocol in a suitable timeframe; thus, arbitrary $\dot{V}$O_2_ max values for firefighters may not be relevant for predicting performance in ARFF-specific tasks, as much of the work is well below the $\dot{V}$O_2max_ [2]. However, further research into aviation firefighting demands is required to confirm this. The findings of Poplin and Roe [20] suggest that this cohort, on average, is in the level 2 fitness category compared to the cohort measured in that study. It should be noted that several firefighters were also fitter than the highest fitness level group reported. While this is largely encouraging, it does suggest that aerobic fitness should still be improved in this group. The caveat is that the type of firefighting in the current study compared to Poplin and Roe [20] is different, i.e., aviation rescue versus metropolitan. Additionally, cardiorespiratory requirements may be less important in firefighting roles associated with low external load, further highlighting the importance of defining specific firefighting roles in research. The maximum $\dot{V}$O_2_ consumption during the scenario was 35.2 mL·kg·min^−1^, equating to 78% of the participants’ predicted $\dot{V}$O_2max_, consistent with values obtained in a run, rescue, exit, and search task (80%) [13] and a six-floor stair climb and six-patient rescue task (83%) [14]. A recent report from the United States looked at firefighter injuries; overexertion and strain accounted for 31% of the 65,650 injuries studied [39]. While this report did not explicitly define overexertion, it can be considered that the cause of injury was a result of firefighters’ physical capacities exceeding the demands of the job. The current research is helpful in starting to quantify the job demands which can be used to help train and select firefighters to match the task demands. This is supported by the work of Albert and Mittleman [40] who report sudden death from cardiac events, which can be attributed to a lack of regular vigorous activity. This finding suggests adding regular vigorous exercise to the routine of firefighters may help to stave off such events.

As participants were assigned to specific roles within the firefighting scenarios, an unavoidable limitation of the study was the overall small sample size, noting the very niche cohort available for this kind of testing. Since the tasks performed by each role varied, internal and external load measures were grouped based on role. Only one female participant volunteered for research, yet this is reflective of the occupation and in line with previous research [2,13,14,22]. A further limitation was the inability to quantify oxygen consumption during the scenario for all participants. However, HR and RPE were recorded to provide an indication of internal load. Future research should aim to collect oxygen demand data for various roles in aviation firefighting.

## 5. Conclusions

The current study successfully quantified the internal and external load involved in an aviation firefighting scenario. Distance travelled, HR, TRIMP, and RPE were presented for all active firefighting roles, while oxygen consumption was reported specifically for the DR role. This role-specific characterisation has not been previously reported for the aviation firefighting specialty. However, except for distance travelled, there were no significant differences in load variables between firefighting roles. Aviation firefighters displayed HR, RPE, and oxygen consumption values comparable to those presented in previous firefighting studies. However, as most research to date has focused on structural firefighting, this promising initial investigation into quantifying the demands of a niche area highlights the need for additional dedicated research to understand the load placed on aviation firefighters. It is important to assess the internal and external load of this occupation to assist with developing safety regulations, training tasks, and minimum physical requirements. Furthermore, aviation firefighting simulations should be representative of real-life scenarios to provide an accurate assessment of occupational workload. The findings from this research have been discussed in relation to injury prevention with clear evidence supporting the benefit of improving ‘fitness’ levels to protect from injury. To draw a corollary between preparing team sports athletes for the rigours of their game, likewise, using data like those presented here, we have a duty to physically prepare firefighters to ensure the safety of themselves and those that they protect from injury and death every day.

## Figures and Tables

**Table 1:** Scenario testing schedule. Day 1 involved baseline testing, with scenario testing occurring on Day 2 (no days in between). Participants completed the aircraft scenario twice (cold and hot). A ‘cold’ scenario involved no real fire, whereas one spot on the aircraft was lit on fire for the ‘hot’ scenario.

Day	Scenario
1	Anthropometric and demographic data collection
	Baseline testing—CMJ, SJ, IMTP, IPU, and multi-stage fitness test
2	Scenario 1—aircraft (COLD)
	Scenario 2—aircraft (HOT)

CMJ, countermovement jump; SJ, squat jump; IMTP, isometric mid-thigh pull; IPU, isometric push-up.

**Table 2:** Job roles and descriptions for the aircraft firefighting scenario. Participants were placed into a job role, reflecting the role assumed when responding to an incident, not their everyday role in emergency aviation services.

Job Role	Job Description
Firefighter: Driver (DR)	Roll out and position hoses at the beginning of the scenario while the BA team is donning the required additional self-contained breathing apparatus (SCBA). Reposition hoses once charged with water. Once the BA team has entered, DR and OP roles roll out and position backup hoses.
Firefighter: Operator (OP)	Same as DR.
Breathing Apparatus (BA) Team: Leader	Wears an SCBA and enters the aircraft to fight the fire internally. Works as part of the ‘BA team’ and instructs the branch. Additionally, helps to feed the hose to the internal BA branch position.
BA Team: Branch	SCBA wearer who operates the hose within the aircraft. Receives assistance and directional instruction from the BA team leader.

**Table 3:** HR zones and corresponding %HRmax.

Zone	%HRmax
1	50–59
2	60–69
3	70–79
4	80–89
5	>90

%HRmax: percentage of maximal heart rate.

**Table 4:** Physical testing variable results.

Descriptive Measures	N	Median	IQR	Minimum	Maximum
HRmax (bpm)	15	193.00	15.50	169.00	208.00
20 m Multi-Stage Fitness Test Distance (m)	15	1680.00	350.00	1020.00	2660.00
Predicted $\dot{V}$O_2max_ (mL·kg·min^−1^)	15	47.11	5.43	36.56	61.17
CMJ Maximum Jump Height (cm)	16	34.50	7.00	23.70	45.10
SJ Maximum Jump Height (cm)	16	29.70	5.35	21.40	38.90
EUR	16	1.14	0.12	0.97	1.46
IMTP Peak Force (N)	16	3851.50	773.50	2425.00	4891.00
IMTP Peak Force (BW)	16	4.25	1.33	3.10	5.80
IPU Peak Force (N)	16	1175.50	354.25	865.00	1697.00
IPU Peak Force (BW)	16	1.45	0.45	0.90	1.90

HRmax, maximum heart rate; $\dot{V}$O_2max_, maximum oxygen consumption; CMJ, countermovement jump; SJ, squat jump; EUR, eccentric utilisation ratio; IMTP, isometric mid-thigh pull; BW, body weight; IPU, isometric push-up; IQR, interquartile range.

**Table 5:** $\dot{V}$O_2_ consumption for DR firefighters in the aviation scenario.

VO_2_ Consumption Measures	N	Median	IQR
Average Oxygen Consumption (mL·kg·min^−1^)	7	19.5	4.70
Average Oxygen Consumption (%max)	7	49	10.50
Maximal Oxygen Consumption (mL·kg·min^−1^)	7	35.2	6.55
Maximal Oxygen Consumption (%max)	7	78	8.00

## Data Availability

Data may be made available to individuals depending on the specific requirement. It should be noted that contractual agreements may prohibit the release.
